# Fetal skeletal dysplasias: a new way to look at them

**DOI:** 10.1590/0100-3984.2018.0140

**Published:** 2020

**Authors:** Miguel Pereira Macedo, Heron Werner, Edward Araujo Júnior

**Affiliations:** 1 Unit of Experimental Biology - Department of Biomedicine Center for Medical Research, Faculty of Medicine, University of Porto, Porto, Portugal.; 2 Department of Radiology, Clínica de Diagnóstico por Imagem (CDPI), Rio de Janeiro, RJ, Brazil.; 3 Department of Obstetrics, Escola Paulista de Medicina da Universidade Federal de São Paulo (EPM-Unifesp), São Paulo, SP, Brazil.; 4 Medical Course, Universidade Municipal de São Caetano do Sul (USCS), Campus Bela Vista, São Paulo, SP, Brazil.

Skeletal dysplasias are a heterogeneous group of over 450 genetic diseases affecting bone and cartilage. They have an incidence of 2/10,000 live births and a lethality of approximately 50%, which makes their prenatal diagnosis of particular importance in determining fetal outcomes as well as in genetic counseling for future pregnancies(^1^).

**Figure 1:**
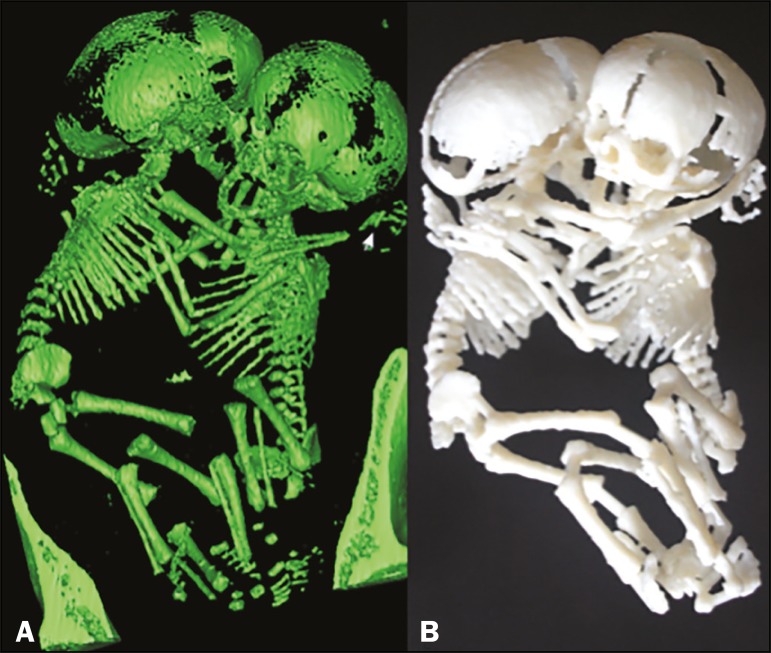
Thoraco-omphalopagus conjoined twins at 31 weeks of gestation. A: 3D view from a CT scan. B: 3D-printed model.

**Figure 2:**
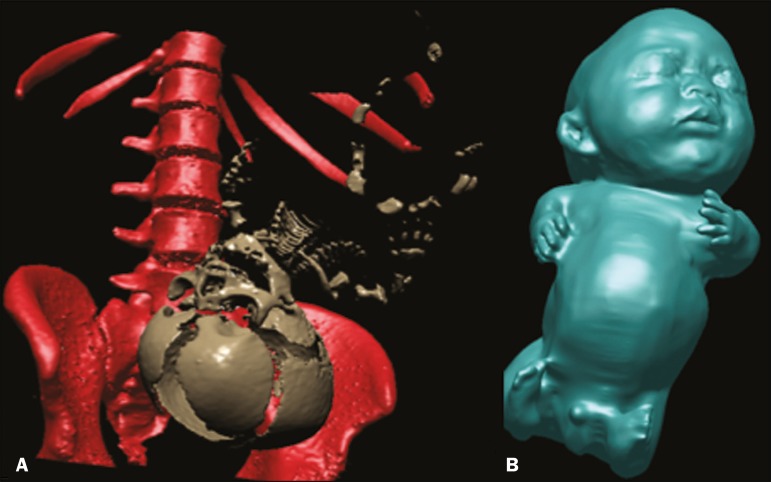
Achondrogenesis at 34 weeks of gestation. A: 3D view from a CT scan. B: 3D view from an MRI scan.

Ultrasound has for many years been the preferred method of screening for these conditions. Despite significant advances in the last decades regarding image quality and acuity, the diagnostic success rate remains disappointingly low, a correct ultrasound diagnosis being made in only 67.9% of cases(^2^). Therefore, new diagnostic modalities should be considered to maximize morphological information. Magnetic resonance imaging (MRI) and computed tomography (CT) have both proven to be useful adjuncts in these conditions. Both techniques have the advantage of being operator independent and not being limited by maternal body mass index or the presence of oligohydramnios. Because it allows three-dimensional (3D) reconstruction, CT has proven to be a valuable complement in selected cases of fetal skeletal dysplasia in which the specific diagnosis cannot be made by ultrasound alone. It has been shown to be superior to ultrasound for the evaluation of bone abnormalities, especially those involving the skull, ribs, or pelvic bones(^3^). The diagnostic gain comes from an appreciation of the morphology and deformities in their entirety. Although the use of CT is controversial because it exposes patients to radiation, it has not been shown to increase the occurrence of malformations; if used judiciously, its benefits may well outnumber its risks(^4^). Regarding safety, unenhanced MRI (i.e., that performed without contrast) has not been associated with teratogenic or adverse fetal effects. However, its use is currently not recommended before 22 weeks of gestation, and, even thereafter, it should be performed in 1.5 T scanners. The initial MRI evaluation of congenital anomalies should include T1- and T2-weighted sequences. If musculoskeletal disorders are suspected, echo-planar imaging, thick-slab T2-weighted sequences, dynamic steady-state free precession sequences, and volumetric interpolated breath-hold examination sequences should be performed(^5^). T2-weighted MRI appears particularly useful in the study of the fetal brain, as well as in that of organs with high water content, which should be evaluated with a T2-weighted half-Fourier single-shot turbo spin echo protocol(^6^). For limb deformities, clubfoot, and arthrogryposis, 3D images obtained through thick-slab T2-weighted sequences generate easily recognizable images of the deformities with the additional advantage of the shine-through effect. Data collected with these techniques allow 3D reconstructions to be modeled into virtual reality constructs or 3D-printed representations (Figures 1 and 2), both of which are useful tools for fetal surgeons who are preparing to perform in utero or early postnatal corrective surgery(^7^). Such imaging techniques can also give parents a greater, more tangible understanding of the defect, as well as strengthening the maternal-fetal attachment(^8^).

Ultrasound continues to be an invaluable tool in the screening and diagnosis of fetal skeletal dysplasias and will not be replaced by either CT or MRI used in isolation. Rather, these serve as complementary imaging techniques that increase the overall sensitivity and specificity of the analysis, allowing a greater number of correct and early prenatal diagnoses.
